# The human microbiome project at ten years - some critical comments and reflections on “our third genome”, the human virome

**DOI:** 10.20517/mrr.2022.20

**Published:** 2023-03-29

**Authors:** Harald Brüssow

**Affiliations:** KU Leuven, Department of Biosystems, Laboratory of Gene Technology, Leuven B-3001, Belgium.

**Keywords:** Microbiome, disease, association, causation postulates, fecal transplantation, probiotics, bacteriophages, virome

## Abstract

The Human Microbiome Project (HMP) has raised great expectations claiming the far-reaching influence of the microbiome on human health and disease ranging from obesity and malnutrition to effects going well beyond the gut. So far, with the notable exception of fecal microbiota transplantation in *Clostridioides difficile* infection, practical application of microbiome intervention has only achieved modest clinical effects. It is argued here that we need criteria for the link between microbiome and disease modelled on the links between pathogens and infectious disease in Koch’s postulates. The most important question is whether the microbiome change is a cause of the given disease or a consequence of a pathology leading to disease where the microbiome change is only a parallel event without a causal connection to the disease – in philosophical parlance, an epiphenomenon. Also discussed here is whether human virome research is a necessary complement to the microbiome project with a high potential for practical applications.

## MICROBIOME

### Quo vadis HMP? 

Ten years ago, a landmark paper was published by the Human Microbiome Project Consortium. It stated that even healthy individuals differ remarkably in their microbes, with strong niche specialization, when analyzing 18 different body sites including the oral cavity, the gut, skin and the vagina in 300 individuals^[1]^. When marking this anniversary, Ruth Ley, one of the pioneers of this initiative, showcased the knowledge gained over the last decade in the microbiome field. The published data demonstrate that the microbiome is key to the proper functioning of our bodies ranging from steering the maturation of our developing immune system to aiding the digestion of food, and counterbalancing pathogens. Distinct microbe compositions in their human carriers were linked to common illnesses such as cardiovascular disease and obesity^[2]^. Ruth Ley wondered why we outsource (sic!) so many important functions to the microbes that we pick up from our environment. It might be better to speak of “attributing” rather than “outsourcing” so many important functions to commensal microbes. Whatever term is used, they seem to express some doubts about the logical plausibility of such an assumption. As a next step, she asked for more sequencing of microbiomes from more diverse human populations, and also from feral animals in order to place our own species’ data in the context of the tree of life. 

**Figure 1:**
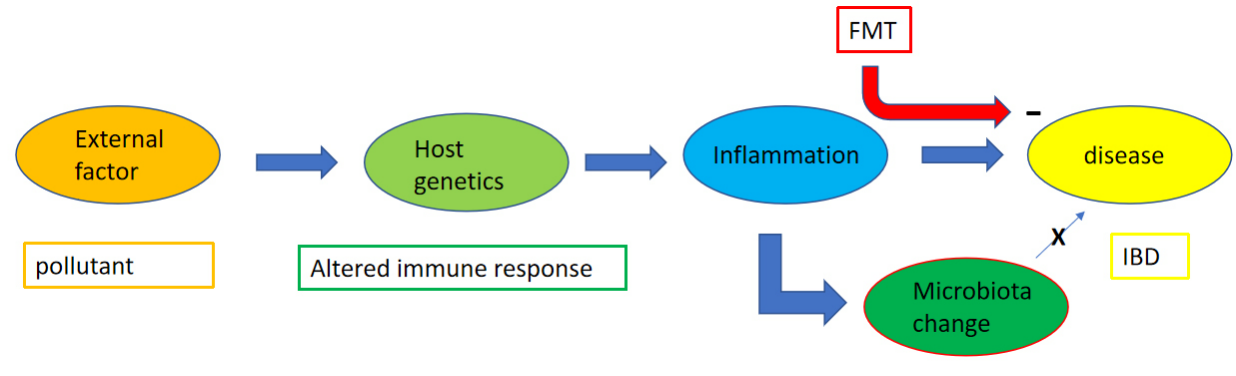
Proposal of a theoretical flow scheme where a microbiome change (“epiphenomenon”) is a consequence of pathogenic mechanisms, but not a cause of the disease. The scheme is considered to be generic, but one might think of IBD. See text for details. FMT: fecal microbiota transplantation; IBD: inflammatory bowel disease IBD.

**Figure 2:**
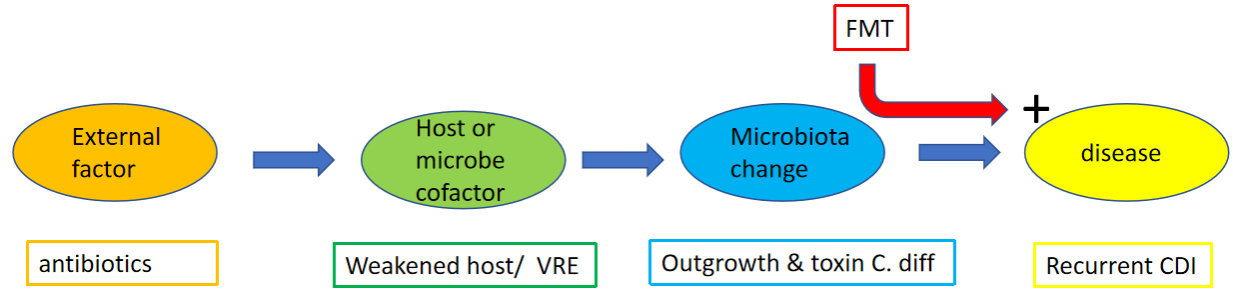
Proposal of a theoretical flow scheme where a microbiome change (“phenomenon”) is in a causal relationship with a disease. The example is recurrent *C. difficile* infection (CDI). VRE: vancomycin-resistant *Enterococcus*. See text for details. CDI: Clostridioides difficile infection; FMT: fecal microbiota transplantation;

One might argue that after a decade of intensive human microbiome inquiry (which filled the columns of the best scientific journals), there are questions that are more urgent than only extending the descriptive part to a larger fishing expedition using a wider net. Now it might be time to assess the acquired knowledge and ask a few critical questions. For example: What are the distinct human phenotypes associated with this “second” genome? A skeptical observer might also ask whether we can expect clear human-associated phenotypes if the microbiome varies so much between individuals. How many links are mere associations not backed by mechanistic data in animal models? How much of the studies on animals could be translated into studies on humans? The old dictum applies here too, that associations are not yet causations. In one case, the microbiome change is in a parallel line, causally unconnected to disease but the consequence of factors leading to disease (microbiome as an epiphenomenon in philosophical speaking) [Figure 1]. In another case, the microbiome change is in a direct chain of cause and effects towards a disease state (microbiome as a phenomenon) [Figure 2]. Whether the microbiome change is an epiphenomenon (consequence of a disease state) or else a cause for a disease can be tested. Consider an intervention that changes the microbiome composition (using substrates, prebiotics, probiotics, antibodies, phages or fecal microbiota transplantation, to name some of the possibilities) and observe whether this change has an effect on the disease condition [Figures 1 and 2]. Of course, this implicates clinical trials that are time-consuming and costly. In addition, if a microbiome change has irreversibly precipitated a pathological chain reaction towards disease, a later correction of the microbiome might not alleviate the disease state any longer. Then you might still have the possibility of prophylactic interventions to prevent the development of a microbial “dysbiosis” in the first place. However, the use of the term “dysbiosis” is debatable since it lacks a precise scientific definition^[3]^. “Dysbiosis” can refer to a change in the composition of a bacterial community; a change in metabolites produced by a bacterial ecosystem; a gross increase of a bacterial load at a given body site; or a shift in colonization to an unusual anatomical site. In view of the physiological variability in the microbiota composition, even among healthy subjects, some variances in microbiota composition are expected in case-control studies using a small number of subjects. So far, causality linking “dysbiosis” to disease has not been demonstrated in many instances. Therefore one might prefer to avoid the umbrella term “dysbiosis”. To highlight the undefined character of the term “dysbiosis”, it is noted in the current text in quotation marks. Strikingly, the microbiota literature abounds with reports that link disease states with microbial “dysbiosis” while few studies expressively try to refute the link between microbiota “dysbiosis” and disease. This bias in microbiota literature is critical since, according to arguments from the philosopher Karl Popper, scientific progress to better theories lives from conjectures and particularly from trials to their refutation^[4]^.

To come back to prophylactic interventions: this concept likewise needs verification by clinical trials which are even more time-consuming and costly than treatment trials. Despite these practical and theoretical difficulties, the maturity of a scientific field is defined by its practical applications (however, here, the author may reveal his industrial research background and his activity as an editor of a biotechnology journal). A famous philosophical dictum says, “Philosophers have only interpreted the world in various ways. The point, however, is to change it”, which certainly also applies to microbiome research. 

### RCT as test bed: treatment of malnutrition with microbiome-targeted nutritional interventions

Several randomized controlled clinical trials (RCT) with gut microbiome interventions led by Jeffrey Gordon have been conducted in the field of childhood malnutrition. The basic microbiome observation was made with children from Bangladesh who suffered from severe acute malnutrition (SAM), defined as having weight-for-length measurements that are more than 3 standard deviations below the median of the reference population, i.e. they had a WLZ score of -3. These children lagged in the development of their gut microbiota composition changes behind that of local, healthy control children^[5,6]^. Subsequently, researchers, in collaboration with J. Gordon, identified in gnotobiotic mice some complementary food ingredients that selectively increased the representation of microbes characteristic for the weaning phase. These age-discriminatory bacteria in the gut microbiota were deficient in malnourished children. Based on these insights, several “microbiota-directed complementary food” (MDCF) items differing in composition (containing chickpea flour, soy flour, peanut flour, and banana as in MDCF-2, or only two of these ingredients) were tested for growth effects in gnotobiotic piglets. Better weight increases were observed with the 4- compared to the 2-ingredient formulation. Then, three different MDCF formulas and a commercially available, ready-to-use supplementary food (RUSF, which is rice- and lentil-based) were tested in Bangladeshi children suffering from moderate acute malnutrition (MAM). After 1 month of treatment, all three MDCFs and the RUSF control group improved WLZ scores from -2.2 to -1.9, with no difference between the groups. However, MDCF-2 produced a significantly greater increase in mid-upper arm circumference (MUAC): a 2.3% increase compared to a 1.6% increase observed with RUSF^[7]^. Since only 14 to 17 children were recruited per group, the ability to detect differences was, therefore, low. In a subsequent RCT, 123 Bangladeshi children with MAM were randomized to a 3-month treatment with MDCF-2 or RUSF. The WLZ scores ameliorated moderately in both groups (again from about WLZ -2.2 to -1.9), but the amelioration was statistically better with MDCF-2 than with RUSF, although the difference was small. At the end of the treatment, the difference in MUAC increase between both groups was statistically not significant. The researchers conducted a sophisticated plasma proteome analysis which revealed a positive association between WLZ amelioration and biomarkers for bone growth and nerve development in the MDCF-2 group. The gut microbiome analysis revealed a negative association between WLZ score ameliorations with two bacterial taxa in the gut microbiome (*Bifidobacterium longum* and *Escherichia coli*) while, for example, *Prevotella* showed a positive association^[8]^. This conclusion is surprising since *B. longum* has been associated with numerous beneficial results in breastfed infants, while the level of this organism was negatively associated with the ponderal growth rate in the children treated with this nutritional intervention. An independent two-year longitudinal study of 222 healthy Bangladeshi children came to a contradictory conclusion. In this study, *B. longum* was positively and *Prevotella* was negatively associated with weight-for-length and weight-for-age Z scores^[9]^. While moderately malnourished and control children from Bangladesh cannot be compared directly, it is nevertheless uncomfortable that two analytically highly sophisticated studies come to such divergent conclusions with respect to the growth-influencing role of gut microbiota taxa in children from a single, particularly well-investigated population. The second study^[9]^ also reported strong seasonal shifts in the gut microbiome composition for 39 bacterial species, which will additionally complicate the establishment of reproducible microbiota-growth associations. 

The first report^[8]^ has been criticized for its small nutritional improvement, which is clinically not significant^[10]^. The outcome contrasts sharply with data reported by another study^[7]^ on the treatment of 343 Bangladeshi children suffering from SAM, treated after appropriate antibiotic application with a rice-lentil formulation, a chickpea-containing formulation (both locally produced)^[11]^, or a ready-to-use therapeutic food (RUTF: Plumpy’Nut, a commercial peanut butter preparation). With these therapeutic formulas, WLZ scores decreased during hospitalization from -3.5 to -2.0 at discharge from the hospital, a much larger effect than achieved with MDCF-2 in children with MAM. One might ask whether it is justified to compare nutritional interventions in MAM and SAM and a follow-up of children in a birth cohort study. It is not clear whether MAM is easier to treat with a nutritional intervention than SAM^[12]^. Due to a greater expected effect size, it might have been easier to conduct a microbiome-anthropometry correlation study in infants with SAM than with MAM. The small effect size in the MAM trial might partly explain the discrepant conclusions between^[8]^and^[9]^. 

### Development of postulates for causality in microbiome-disease links

The observation that a “microbiota-directed complementary food” (MDCF) had, in two clinical trials, only a small treatment effect on MAM of borderline clinical benefit might suggest to a skeptical reader that the gut microbiome is more likely a consequence of factors that lead to malnutrition-hence an epiphenomenon- than a cause of malnutrition (see scheme in Figure 1). As postulates have been developed that must be fulfilled to link a pathogen with a disease (Koch’s postulates and its derivatives^[13]^), adapted postulates have to be developed for linking specific gut microbiome constellations with the disease. As microbiome changes have frequently been associated with health changes, the difficult definition of health and its measurement^[14]^is a further complication when defining microbiome-health links. Statistical associations are probably not enough, even when combined with experimental work in gnotobiotic mice. Without more medically oriented “microbiome-disease postulates”, the microbiome field runs the risk of raising high hopes that are not backed up in medical practice. This could ultimately disappoint grant agencies and investors. To quote an old dictum of the Latin poet Horace Parturient montes, nascetur ridiculus mus (the mountain laboured and brought forth a mouse).

**Figure 3:**
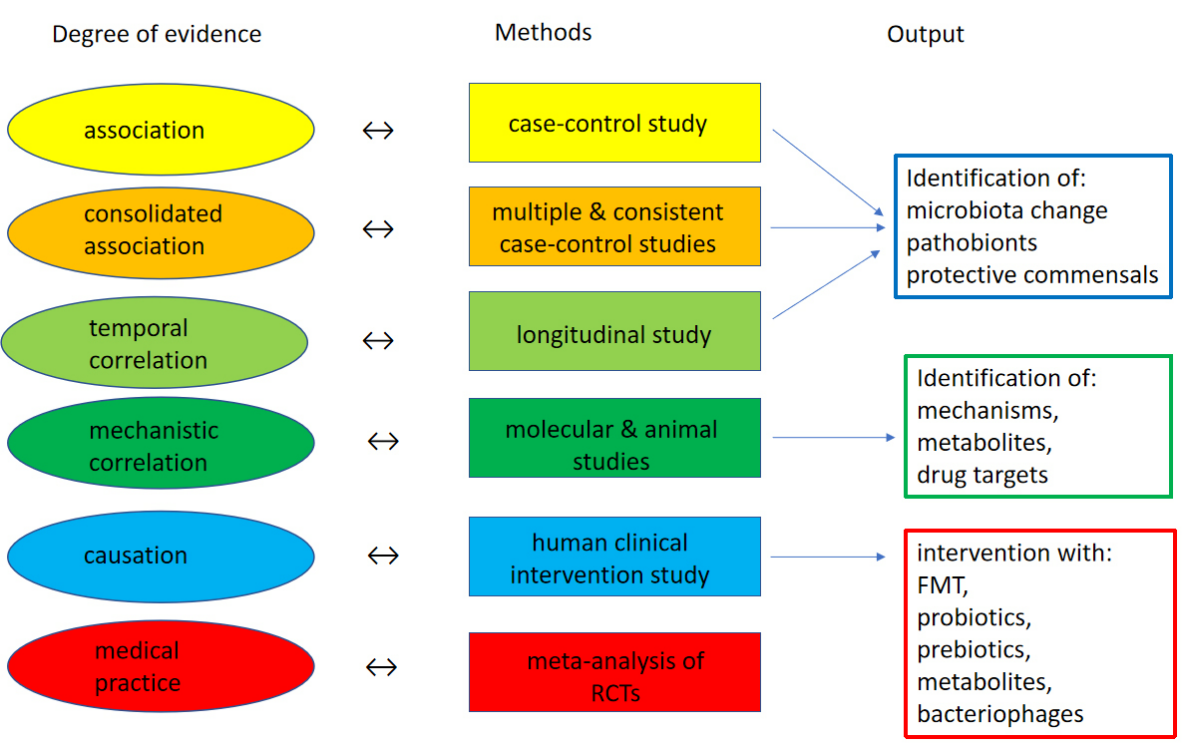
Proposal of postulates for establishing a causal link between microbiome change (microbiota “dysbiosis”) and disease. See text for details. FMT: fecal microbiota transplantation; RCT: randomized controlled clinical trials.

While it is currently over-ambitious to develop Koch postulate-like criteria for microbiome-health links, it might be useful to discuss a tentative set of rules [Figure 3]. One might speak of *associations* when case-control studies demonstrate statistically significant microbiome differences between patients of a specific disease and matched healthy controls. One might speak of *consolidated associations* when this difference has been consistently confirmed in independent studies from several geographical areas. At the next level, one might speak of *temporal correlations* when longitudinal targeted population studies show that the identified microbiome changes do not follow, but precede the disease manifestation. At this level, it is desirable that a microbiologically defined complex of disease-associated “pathobiont(s) or health-associated “benebionts(s) (to invent a tentative term) can be identified instead of a purely statistical term. Bolstering the argument would be the evidence that the transplantation of the specifically changed microbiome from patients reproduces relevant pathological aspects of the human disease in an appropriate animal model. Alternatively, one might require that a suspected pathobiont from the altered microbiome displays genes/characteristics explaining aspects of the pathology in the patients or reproducing some pathological aspects of the disease in animals. Evidence would be needed that the suspected beneficial commensal secretes compounds that stabilize the microbiome, inhibit the pathobiont *in vitro* and in vivo and protect the host against disease. If these criteria are fulfilled, one might say that *mechanistic correlations* have been met. Fulfillment of these sequential criteria is, however, not yet sufficient to establish a causal microbiome-disease link. For that proof, it needs a human study that satisfies the criteria of a RCT where a targeted intervention based on the insights of the microbiome analysis leads to a clinical amelioration in affected patients or prevents disease development in subjects at risk of this disease. Interventions might occur by FMT; probiotic / beneficial commensal; specific prebiotics supporting the beneficial commensal; synbiotic combinations; bacteriocins; or phages targeting the identified pathobiont. Only when intervention trials provide positive signals may one speak of *causality* between microbiome change and disease. Of course, these postulates set the bar very high for going from association to causation. However, similar high standards are required to causally link pathogens to infectious diseases. It is not evident why comparably strict criteria should not be requested for causality in the microbiome-disease field. Without a set of strict criteria, associations from small case-control studies are too lightly accepted as causation, leading to spurious links. This would raise false hopes with respect to the prospect of intervention studies.

In the following passages, the argument depicted in Figures 1 and 2 will be illustrated with data from clinical trials about a clear-cut microbiome intervention: namely fecal microbiota transplantation (FMT). 

### Fecal microbiota transplantation works very well in CDI…. 

FMT is commonly cited as a breakthrough clinical application in gut microbiota research and the established case is *Clostridioides difficile* infection (CDI). Case series, many of them already conducted before the rise of microbiome research^[15]^, demonstrated that the infusion of intestinal microorganisms in the form of a fresh bacterial suspension of healthy donor stool into the intestine of 317 CDI patients restored the microbiota balance and resolved the symptoms in 92% of CDI patients without causing major adverse events. This, in the context of clinical research and practice, is a remarkable success rate. A large, randomized controlled clinical trial (RCT) comparing fresh versus frozen FMT in 219 CDI patients concurred with the outcome of the case studies and showed resolution of symptoms in 70% and 75%, respectively^[16]^. Researchers considered two possibilities to explain the efficacy of FMT in CDI patients: either the patients had completely lost their own healthy microbiota and needed provision of these strains or their microbiota were unable to reestablish the equilibrium. In the first case, FMT would provide the missing beneficial bacteria. In the second, FMT would provide missing non-bacterial factors that help to reestablish the equilibrium of the bacterial population. Two clinical reports might shed some light on this question. In one study, 46 patients with recurrent CDI received either healthy donor (heterologous) stool or their own (autologous) stool. The cure rate was very high in the heterologous stool transplantation group (91%), but at 63%, still astonishingly high in the autologous FMT group^[17]^. This small trial asks: what was provided with the autologous stool? The second report is from a small clinical case series of 5 patients with chronic relapsing CDI who received fecal filtrate transfer (FFT), i.e., a donor stool lacking viable bacteria. Interestingly, FFT also restored normal stool habits and resolved symptoms of CDI over a minimum follow-up period of 6 months^[18]^. One should not draw far-reaching conclusions from such a small number of cases. In addition, a cell-free stool fraction may contain many compounds (bacterial metabolites, bacteriocins, host metabolites and viruses). The role of metabolites in the efficacy of FMT, sometimes called the dark matter in FMT, has not been systematically investigated^[19]^. So far, mouse experiments showed that oral butyrate reduced inflammation and mucosal damage and that oral secondary bile acids reduced the severity of dextran sodium sulfate (DSS)-induced colitis. The small case series^[18]^ studied only viruses. The donor virome in that study (only one donor-patient stool pair could be analyzed) was dominated by a variety of *Lactococcus *bacteriophages and it induced, in the single analyzed recipient, a fecal virome shift towards more *Lactococcus *phages and a decrease of enterobacterial phages. A subsequent longitudinal fecal virome study of 24 CDI patients treated with FMT, or the antibiotic vancomycin compared to 20 untreated household controls, described both a microbial and a viral “dysbiosis” in CDI patients. In treatment responders, FMT was associated with alterations in both the virome and the bacterial microbiome^[20]^. Other factors certainly play pathological roles in the development of CDI such as metabolic interactions between enterococci and *C. difficile*, which stimulate toxin production by the latter^[21]^. Whatever the final explanation of the mechanism of FMT on CDI may be, its high efficacy, compared to the modest success of antibiotic treatment^[22]^, proves a causative link between gut microbiome “dysbiosis” and CDI along a line depicted in the scheme of Figure 2. 

### …but less so in IBD

The high success rate of FMT could not be repeated in other medical conditions for which gut microbiota imbalances were described. An example is ulcerative colitis (UC), a subtype of inflammatory bowel disease (IBD). One RCT with 75 patients reported remission in 24% of FMT recipients compared to a 5% remission rate in placebo (water) recipients^[23]^. In a second RCT with 40 UC patients, a 30% and 20% remission rate was seen with heterologous and autologous FMT, respectively^[24]^. In a third RCT with 80 UC patients receiving a weekly healthy donor FMT or placebo for two months, remission rates were 27% and 8%, respectively^[25]^. In a fourth RCT with 73 UC patients receiving anaerobically prepared heterologous or autologous FMT, remission rates were 32% and 9%, respectively^[26]^. From these clinical data, it seems that only a quarter of UC patients benefit from healthy donor FMT, while 5% to 8% of patients benefit from placebo and 9% to 20% from FMT with their own stool. In one study^[24]^, the difference between heterologous and autologous FMT was statistically not significant. Even when not accounting for the amelioration in the control patients, three-quarters of the UC patients receiving FMT experienced no beneficial effect, which suggests a situation in which the gut microbiota is an epiphenomenon or a consequence of other factors leading to IBD as conceptualized in the scheme depicted in Figure 1. The effectiveness of FMT in patients with Crohn’s disease (CD), the second clinical manifestation of IBD, remains unclear as current studies reporting potential efficacy are limited in cohort size and lack a placebo treatment group^[27]^. The largest study so far with 19 FMT-treated CD patients showed an amelioration in clinical score and quality of life in 58% of the patients but no change in endoscopic index of severity^[28]^. 

Microbiome “dysbiosis” has been studied in various other clinical conditions such as gut diseases or allergic diseases in children ranging from food allergy to atopic dermatitis^[29]^. Changes in oral microbiome composition were linked with dental diseases (dental caries, periodontal disease); changes in vaginal microbiome composition were associated with bacterial vaginosis; and changes in skin microbiome composition were connected with dermatological diseases (acne, atopic dermatitis). Additionally, some forms of microbiome transplantation have also been tried, e.g., the treatment of bacterial vaginosis with local application of live lactic acid bacteria, sometimes even in the form of plain yoghurt. 

Somewhat related to FMT but based on different concepts are probiotic interventions, i.e., the application of cultured, beneficial, health-promoting bacteria. A large number of microbiological and clinical research has been conducted in this field and might provide an idea of what can be achieved with targeted microbial approaches for medical applications. A well-documented case is about necrotizing enterocolitis (NEC) in very preterm and very low-birthweight infants. A Cochrane review has analyzed probiotic interventions (mostly with bifidobacteria and lactobacilli) in 11,000 cases of preterm infants enrolled in 56 clinical trials^[30]^. Meta-analysis showed that probiotic interventions may reduce the risk of NEC (relative risk (RR) of 0.54 compared to placebo), mortality (RR 0.76), and late invasive infection (RR 0.89). The evidence was judged to be of low or moderate certainty. Funnel plot analysis revealed a publication bias from studies with small numbers of cases that over-reported positive outcomes with probiotics. Another Cochrane review analyzed synbiotic (probiotic-prebiotic combinations) interventions in the cases of 925 very preterm infants enrolled in six clinical trials^[31]^. Meta-analysis suggested that synbiotics may reduce the risk of NEC by an impressive rate (RR 0.18) and may decrease mortality (RR 0.53), but not late-onset invasive infections. However, the trial data provided only low-certainty evidence for the efficacy of synbiotics. The reviewers concluded that confirmation is needed in the form of large, high-quality trials. Such a trial has indeed been conducted in 4,556 newborns from rural India. The infants had a mean birth weight of 2.7 kg and were thus not at risk of NEC. The newborns received a synbiotic (*Lactobacillus plantarum* plus fructooligosaccharide) or a placebo. About 300 physician-diagnosed cases of sepsis were observed, and the researchers detected a 40% sepsis reduction by the synbiotic. Mortality did not differ between the two groups (Panigrahi *et al*., 2017)^[32]^. 

Probiotic effects were also reported for atopic dermatitis (AD). While numerous small studies reported promising results^[29]^, a Cochrane review on probiotic treatment (predominantly with bifidobacteria and lactobacilli), mostly in pediatric AD cases from 39 trials comprising 2,600 patients, showed no clinically significant effect of probiotics on participant-rated eczema symptoms or quality of life. Physicians noted a slight reduction in eczema scores, but the difference was not clinically significant. The authors concluded that the use of probiotics for the treatment of eczema is currently not evidence-based^[33]^. Another systematic review came to a more optimistic conclusion when exploring the effect of a specific probiotic, Lactobacillus LGG, applied in the perinatal period on the development of AD. They evaluated 11 trials which enrolled a total of 2,600 infants. Overall, a significant risk reduction of 35% was found^[34]^.

The striking success of FMT in CDI might have caused some over-enthusiasm for this approach with respect to other diseases. The variable outcome of clinical trials with probiotics conducted over the last two decades should serve as a reminder that it is a long way from a microbiological concept to an evidence-based medical treatment.

## VIROME

In the second part of this perspective, I focus on a somewhat neglected aspect of the human microbiome, namely the human virome. Part of the neglect is due to technical problems. Unlike the 16S rRNA genes shared by bacteria and archaea, no shared marker gene exists for viruses. The analysis of the virome has had to wait for the development of metagenome sequencing technologies. In addition, the sequence database for viral genomes was much smaller than that of bacterial genomes, which makes bioinformatic analyses more complicated. However, the human virome is not only a relatively neglected part of the HMP, but, in my view, its analysis opens substantial possibilities, not only for microbiome engineering but it also holds potential for targeted medical approaches against antibiotic-resistant bacterial pathogens. In the following, I will first look into the role of viruses in IBD, then highlight the role of bacterial viruses (phages) in human health, and finally close with some glimpses into virome studies with high public health importance.

### Virome research in IBD

So far, it is not known why only about a quarter of UC patients responded to FMT with symptom amelioration. As viruses are “our third genome”, looming behind the bacterial “second genome”, researchers turned to virome analyses in IBD to gain complementary insights into the pathogenesis of IBD beyond microbiota, genetics, immunological and environmental risk factors. A comprehensive study was published^[35]^. The researchers started with metagenome sequencing of stool filtrates for 18 Crohn’s disease (CD) and 42 UC patients and compared them with stools from 12 non-IBD household controls. Phages of the *Caudovirales* (tailed double-stranded(ds)DNA phages) order and *Microviridae* (small capsid single-stranded (ss)DNA phages) family were the most abundant viral taxa in stools with less than 5% of other viral sequences. The remaining viruses comprised *Virgaviridae* (ssRNA plant viruses), *Tymovirales* (a morphologically distinct group of ssRNA plant viruses), ds RNA viruses (containing animal reoviruses, bacterial cystoviruses as well as plant, fungal and protozoal viruses), and *Anelloviridae* (circular ssDNA animal viruses of wide distribution, but without disease attribution). 

Plant and insect viruses in the stool were not specific to UC but were frequently reported in human gut virome analyses and probably represent viruses associated with plant material in our food^[36]^. In fact, the bladder virome lacks plant and insect viruses^[37]^. This indicates a general problem: the gut content is external to the human body. It represents food material (with some host secretions) that transits the gut in order to be digested. Therefore, not all of what is found in stool will be relevant for human physiology beyond serving as a source of organic matter and energy for our body. It is therefore questionable whether or not the observation that algal viruses are overrepresented in the gut mucosa from UC patients indicates a dietary risk factor for UC, as suggested by some authors^[38]^. The argument applies *ceteris paribus* to bacteria in the gut microbiota which includes microbes associated with food, particularly with fermented food. This argument might serve as a warning that not everything in the stool makes biological sense for the human host. 

In the study by Norman *et al.*^[35]^, *Caudovirales* showed a significant enrichment over *Microviridae* in UC patients when compared to both CD patients and controls. In the next step, they did metagenome sequencing with the isolated virus-like particle (VLP) fraction from feces. In a longitudinal stool survey conducted on patients and controls, they observed an increase in the richness of *Caudovirales* in IBD patients. Each disease type (CD, UC) harbored unique phages. *Lactococcus, Lactobacillus, Clostridium, Enterococcus*, and *Streptococcus* tailed phages were associated with disease. The stools of CD and UC patients showed a significant reduction in bacterial diversity compared to controls. Phage expansion was not the result of increases in their bacterial hosts. The data from the UK cohorts were reproduced with data from the US cohorts. Within eukaryotic viruses, *Anelloviridae *were more prevalent in IBD patients than in controls but did not correlate with disease activity. 

Another study^[39]^ investigated the viral community in 20 individuals with active UC prior to and four weeks after receiving FMT. Compared to patients who did not respond to therapy, patients who had a clinical response to FMT had a lower relative abundance of *Caudovirales* bacteriophages at the time of transplant. In addition, the relative abundance of *Caudovirales* in non-responders increased after FMT, while no change was observed in responders. The authors suspected that colitis-associated bacteriophages can induce IFN-γ-producing T cells. By *in vitro* experiments, they could indeed show that VLPs from active UC but not inactive UC or healthy controls caused activation of naive CD4^+^ T cells and potent induction of IFN-γ. 

Additionally, other researchers^[40]^ did a bioinformatic reanalysis of the dataset in^[35]^. They argued that since the virome displays enormous diversity and inter-individual variation (in one study, even identical twins differed in their virome^[41]^), it is essential to analyze data at a higher viral taxonomical level (i.e., at the protein homology level) in order to detect trends. They noted^[40]^ that none of the three key viral clusters (VCs) which were associated with the core virome in healthy individuals featured genes associated with lysogeny, while all but one of the seven key VCs associated with IBD also featured lysogeny genes. The observation that lytic phages predominate in the stool of healthy subjects and temperate phages in the stool of diseased patients cannot be generalized beyond IBD. In fact, two studies conducted 40 years apart showed not only an increase in phage titer but also an increase in the proportion of virulent coliphages in the stools of Japanese patients compared to healthy controls^[42]^. This was also observed when comparing stool phages in healthy children and pediatric diarrhea patients from Bangladesh. In the latter case, 95% of the phages isolated from diarrhea patients were virulent phages, mostly T4-like coliphages^[43]^. Temperate phages were also the most prevalent coliphage isolates in the stool of 1-year-old healthy children from a more recent study^[44]^. 

Clooney *et al.*^[40]^ observed that alterations in the gut virome of IBD patients occurred in conjunction with changes in the bacteriome. They suggested a model where environmental stressors associated with the inflamed gut, such as reactive oxygen species (ROS), could induce prophage in lysogenic bacteria to enter the lytic cycle. This would cause a reduction in the affected bacterial species, resulting in a decrease in bacterial diversity and an increase in temperate *Caudovirales*. This interpretation concurs with the observation that IBD-associated phages were predominantly tailed phages, which contain many temperate phage members, while the gut virome of healthy subjects was dominated by virulent *Microviridae*. 

Stool is a convenience sample for gut microbiome analyses, but the question was raised whether stool is a reliable indicator of the microbiome in the gut. Therefore some researchers^[38]^ worked with tissue biopsies from the rectum instead of stool as source material for their analyses. Samples were obtained from 63 UC patients and 48 healthy controls from Hong Kong. *Caudovirales* abundance was significantly higher in UC, while viral species diversity was decreased compared to controls. Notably, no difference in the diversity of mucosal virome was observed between non-inflamed mucosa of patients with UC and healthy control mucosa, while differences were detected between inflamed UC mucosa and controls. Virome “dysbiosis” in UC is a highly individual patient trait, and when using two reference groups from mainland China, the researchers detected geographical effects on the mucosal virome structure. At the genus level, *Ascovirus* (insect viruses) and *Streptococcus* phages were the dominant viruses detected in UC mucosa. In their analysis, the viral communities converged into two clusters where the second cluster was nearly exclusively found in UC patients. This cluster contains many phages of Enterobacteria, including such well-known types as phiX174-, P1-, P22-, lambda- and T4-like phages. 

### Bacterial viruses interacting with the mammalian host

The question arises whether bacterial viruses are irrelevant to the human host since phages cannot infect human cells. However, some data were published showing that bacterial viruses might interact with the human host at several levels. Data on direct interaction are still scarce. One group reports that small amounts of DNA from filamentous phages such as phage M13 survived the digestion when fed to mice. Phage DNA fragments were detected in the blood hours after phage DNA feeding. With PCR and by *in situ* DNA hybridization phage DNA was detected in white blood, spleen and liver cells and cloning experiments purportedly showed the integration of phage DNA into a mouse DNA (Schubbert *et al.*)^[45]^. Other geneticists (Science : Can DNA in food find its way into cells? | New Scientist) expressed skepticism towards these experiments, and to my knowledge, these data were not confirmed independently. 

Much better supported are data about filamentous phages, which enhance the virulence of several important bacterial pathogens such as *Vibrio cholerae* and *Pseudomonas aeruginosa* (Pa). For example, wounds infected with Pa containing a filamentous prophage showed delayed wound healing as compared with wounds infected with a prophage-free Pa. Delayed wound healing was associated with filamentous phage release and linked to inhibition of keratinocyte migration into the wound^[46]^. Pa filamentous phage Pf4 acted directly on phagocytes to suppress the intracellular production of tumor necrosis factor (TNF), a cytokine of the immune system, which then inhibits Pa bacterial engulfment and thus delays clearance of the wound infection. Fluorescently labeled Pf4 was internalized by murine phagocytes and localized both in lysosomal vesicles and in the cytosol. Intracellular Pf4 particles triggered TLR3 expression, a pattern recognition receptor of the innate immune system, driving type I interferon production, which in turn inhibited TNF production and, thereby, phagocytosis^[47]^. These data do not prove that Pf4 phage transcribes RNA in the eukaryotic cell, but 50 y-old data reported that 0.2% of the total RNA from human fibroblasts exposed to phage lambda were lambda RNA^[48]^. That filamentous phages can enter and intimately interact with phagocytes should not surprise since filamentous prophages have learned to leave the bacterial host through the bacterial membrane without compromising the bacterial cell integrity. Filamentous phages might, therefore, also cross eukaryotic cell membranes. In fact, genetically modified filamentous phages were reported to cross the otherwise very tight blood-brain barrier in rats after nasal application^[49]^. 

Researchers have recently drawn attention to the fact that phages interact with the immune system by inducing a specific antibody and T cell responses against phages^[50]^. Phages experience endocytosis^[51]^. Some phages such as T4 express immunoglobulin-like domains on their capsids which leads to interaction with mucus in the gut^[52]^. Cell culture experiments showed transcytosis of phages through epithelial cells enabling contact of phages with intracellular compartments of human cells. Additionally, contacts with subepithelial cell layers were shown^[53]^. Treating germ-free mice with bacteriophages led to immune cell expansion in the gut. *Lactobacillus, Escherichia*, and *Bacteroides* phages and phage DNA stimulated IFN-γ via the nucleotide-sensing receptor TLR9^[39]^. Polish scientists draw attention to potential immunomodulatory activities of phages which might become relevant for phage therapy^[54]^.

### Phages increase bacterial virulence

Interaction of phages in human hosts also occurs at another level when considering phage-encoded virulence factors in bacterial pathogens. Phages that increase the virulence of bacterial pathogens are not limited to filamentous phages (*Inoviruses*). This trait is shared by many phages that integrate their genome as a prophage into the bacterial chromosome (temperate phages). Studies have classically shown that *E. coli* strains containing phage lambda as integrated prophage have an *in vitro* growth advantage over strains lacking lambda. Auxiliary lambda genes also confer anti-phagocytic properties to *E. coli* cells reaching the bloodstream. Subsequently, it became clear that prophages play a crucial role in the virulence of many important bacterial pathogens by encoding important bacterial toxins or virulence factors ^[55,56]^). A striking number of prophage genes are directed against the immune system of the mammalian host, starting with factors that interfere with phagocytosis or that paralyze the immune defense by its overactivation with prophage-encoded superantigens. 

The important role of prophages in the development of bacterial virulence has been rationalized by evolutionary arguments. When the phage integrates its DNA into the bacterial chromosome, it benefits passively from the evolutionary success of the lysogenic bacterium. The bacteria exploit phages as versatile gene carriers (prophage-associated lysogenic conversion genes, phages as transducing agents) to accelerate their evolution. There is speculation that this cooperation goes far back in evolution when eukaryotes were still unicellular and fed on bacteria, as amoeba still do nowadays^[57]^. Bacteria carrying prophages that encode toxins that lyse amoeba could not only escape predation but make a living from hunting amoeba. With the evolution of multicellularity in eukaryotes, amoebocytes patrol the body first in sponges and later as phagocytes in higher animals to fend off bacterial invasion. Bacterial pathogenicity might have evolved from such food predator-prey relationships. It would be surprising if this phage-bacterium genetic cooperation is limited to bacterial pathogenicity- one might well expect that prophages play a similarly prominent role in bacterial commensalism when colonizing animals and plants. The gut- and root-localized bacteria and their associated prophage and phage genomes might be a rich source for commensalism-enhancing viral genes. 

### Do phages transfer antibiotic resistance genes? 

The medical interest in phage-mediated gene transfer into bacteria goes beyond pathogenicity factors and extends to the potential transfer of antibiotic resistance genes. The Covid-19 pandemic was an announced public health crisis and so is the antibiotic resistance gene (ARG) increase in major bacterial pathogens. A group of ESKAPE (*Enterococcus faecium*, *Staphylococcus aureus*, *Klebsiella pneumoniae*, *Acinetobacter baumannii*, *Pseudomonas aeruginosa*, and *Enterobacter* spp) organisms already now escape treatment with common antibiotics. It has been predicted that by the middle of this century, about 10 million people will die annually from antibiotic-resistant infectious diseases - more than the 6.5 million confirmed COVID-19 deaths that have been reported up to now to the World Health Organization (WHO). The crisis involving antibiotic use is thus another impending health catastrophe. 

At the moment, researchers do not agree on what role bacteriophages play in catalyzing the antibiotic resistance crisis. Researchers studying individual phage genomes reported that ARG abundance in about 1200 phage genomes was vastly overestimated and that four of the predicted ARGs failed to confer antibiotic resistance in *E. coli* when tested experimentally. These scientists concluded that ARGs are rarely encoded in phages^[58]^. In contrast, viral fraction and viral sequence reads in clinical and environmental samples contain many ARGs. However, only a few ARGs have been found in viral contigs assembled from metagenome reads, with most of these genes lacking effective antibiotic resistance phenotypes^[59]^. However, in another report, the viral fractions in three types of food (chicken, fish, and mussels) were identified as sources of ARG-carrying phage particles. Their ability to infect and propagate in an *E. coli* host was experimentally confirmed after isolation. To assure the phage particle association, the ARG-containing fraction was further purified by CsCl density gradient centrifugation, and DNA outside the capsids was removed enzymatically^[60]^. Whatever will finally be concluded for ARG within phage genomes, classical phage genetics showed that phages capable of generalized transduction can also transfer ARG. A particularly intriguing case was recently demonstrated^[61]^ and called lateral transduction. A peculiar *S. aureus* prophage from the *pac*-site lineage of Siphophages initiates its DNA replication before the integrated prophage DNA is excised from the bacterial chromosome. This process results in a type of “polytene” replication bubble around the prophage origin of replication. Instead of following the conventional rolling circle replication mechanism of excised phage DNA, this still integrated prophage replicates DNA for several hundred kilobases into adjacent bacterial DNA. DNA packaging then starts *in situ* from the integrated prophage and continues with filling phage capsids by the headful packaging mechanism with the replicated DNA. In this way, substantial amounts of bacterial DNA get into phage particles which then transduce bacterial DNA with high efficiency. Similar mechanisms of lateral transduction have now also been identified for the *Salmonella* phage P22^[62]^. 

### Phages as therapeutic agents

Bacteriophages not only present threats to human health, but also represent potential tools against ESKAPE organisms for physicians. In the Soviet Union, phage therapy (PT) was practiced when antibiotics were scarce^[63]^. The rationale is simple: bacteriophage cocktails that infect and lyse a range of bacterial pathogens are given by various application routes in the hope that they eradicate the pathogen from the patient or at least reduce the titer of the pathogen to such an extent that the patient’s immune system can cope with the remaining level of infection. However, with one notable exception, RCT have so far mostly failed to prove the efficacy of PT. Neither a T4 coliphage cocktail given orally nor a commercial Russian phage cocktail showed treatment efficacy against acute bacterial diarrhea in children from Bangladesh. This failure might be attributed to a too low phage titer used or the fact that the *in vivo* pathogen titers remained below the replication threshold of T4 phages^[64]^. Treatment of bladder infections with a commercial Georgian Pyophage cocktail from the Eliava Institute in Tbilisi was not superior to rinsing the bladder with a placebo or antibiotic treatment^[65]^. The authors suggested that the trial was underpowered with respect to the number of treated patients in order to detect differences. A third PT RCT of burn wound patients infected with *Pseudomonas aeruginosa* was seriously underpowered with respect to the number of treated patients and the phage cocktail suffered additionally from stability problems^[66]^. A fourth carefully controlled RCT of external ear canal infections with *Pseudomonas* phages provided some hints for at least transient efficacy, but the evidence is still preliminary^[67]^. So far, the only successful RCT is a prophylaxis trial from 1963, conducted in Tbilisi (Republic of Georgia), demonstrating a significant reduction in shigellosis and *E. coli* diarrhea in children younger than 3 years, treated orally with *Shigella* phages pressed into pills. The placebo-controlled and cleverly randomized trial was conducted on 30’000 children who were followed for 3 months with microbiological and clinical evaluation^[63]^. More encouraging for the prospect of PT against ESKAPE organisms is an increasing number of case reports and case series, some of them accompanied by careful microbiological analyses, which showed a relatively high success rate of resolving infections, albeit frequently requiring prolonged phage treatment. Treatment success was more remarkable since it was also achieved in desperately ill patients, many of them infected with antibiotic-resistant ESKAPE organisms. The literature comprises 14 case series reports (recent examples are ^[68-71]^) and an increasing number of case reports. For ethical reasons, most of the treatments were conducted with both phages and antibiotics, so the encouraging clinical outcomes might therefore be partly the effect of a synergy between phage and antibiotics, putting the pathogens under double selection pressure. It is possible to pre-adapt a therapeutic phage to a pathogen of an individual patient through traditional methods^[72] ^or genetic engineering^[73]^. Pre-adaptation of phage can also decrease bacterial resistance development^[74]^. Through genetic engineering, one can extend the host range of phages by host receptor binding protein shuffling^[75]^. These possibilities add to the versatility of phages as potential therapeutic tools.

### Phages for microbiome engineering? 

A new development is to extend phage therapy to pathobionts, organisms that cause disease in the presence of dysbiotic microbiota or specific genetic or immunological defects in patients. The phage approach offers the possibility of targeted microbiome engineering with a selective intervention acting at species and frequently even at strain-specific levels. In contrast, antibiotics act against entire classes of bacteria including commensals. Even less selective is FMT, which supplies a whole complex microbiome potentially including pathogenic viruses. As proof of the concept, a large research consortium investigated the gut microbiome in 500 IBD patients from different geographical origins and identified *Klebsiella pneumoniae* (Kp) as a putative IBD-associated pathobiont^[76]^, confirming an earlier independent report^[77]^. The consortium then focused on specific Kp strains, which were increased during IBD disease flares. These strains induced an inflammatory response in the colon of mice. The researchers then composed a cocktail of phages against these Kp strains. In antibiotic-pretreated and then Kp colonized mice, the orally applied phages reduced the gut Kp titer by 1000-fold and ameliorated inflammation in a mouse colitis model induced by a gut irritant. In a technical human gut model, the researchers observed a loss of the phage under conditions of low pH gastric simulation. In healthy human volunteers pre-treated with a gastric acid inhibitor (omeprazole), a high oral phage dose (10^10^ infectious phage particles) survived stomach passage and did not cause adverse effects in healthy volunteers^[76]^. Overall, this is a promising approach and the critical issue is whether the identified Kp is a cause of IBD or just a “parallel” epiphenomenon of a pathological condition leading to IBD without having a direct impact on the disease. Scientists who commented on this report praised the impressive scope of the work but asked how the addition of phages would affect an already overpopulated phage ecosystem in IBD and whether a rise in inflammation might occur as a result of further phage-mediated bacterial killing^[78]^.

### Phages as a motor of molecular biology

In the present context, it is worthwhile to recall that studies on phage-bacterium interaction have historically provided the basis for molecular biology. Phage enzymes and bacterial restriction enzymes controlling phage infection made genetic engineering possible and started the molecular biology revolution. The study of phage-bacterium interaction, particularly the genetic aspects of their arms race, continues to provide tools that have revolutionized genetic engineering, as documented by the discovery of the CRISPR-Cas system discovered when studying the interaction of phages and yoghurt starter bacteria in milk fermentation^[79]^. The analysis of the genetic aspects of the arms race between phages and their bacterial hosts continues to provide new genes and proteins for the toolbox of molecular biology ^[80,81]^ underlining the fruitfulness of phage research for contemporary biology. 

### Adapted (beneficial?) human viruses

A survey of the gut virome would not be complete without mentioning human viruses. As human viruses can infect human cells, one might be tempted to conclude that human viruses found in the gut must have a negative effect on human health via the cytopathic effect on human cells. However, this is not necessarily so. Virome studies have revealed viral infections which remained undetected in the past because they caused no or minimal symptoms. Evolutionary arguments frequently maintain that viral pathogenicity is a maladaptation of viruses from cross-infections of an animal source (zoonosis) that destabilizes the human host. SARS-CoV-2 is an example of this concept. The origin of this virus is probably to be found in animals, particularly bats, where coronaviruses cause only minimal or no disease due to long co-evolution with the natural host^[82]^. In humans, SARS-CoV-2 is still maladapted, causing severe disease in specific individuals. Well-adapted viruses are those that replicate efficiently in the host without causing many symptoms. Many virologists think that this is the evolutionary climax situation for viruses. 

The case of *Alleloviruses* (AV) in human stools might be an illustration of a widely distributed but harmless virus in the human stool. Most humans are either chronically infected or continuously re-infected with *Allelovirus*. Infections start early in infants (diaplacental transmission does not seem to occur). AV species richness increases in early infancy and reaches a maximum level at 1 year of age. Over 100 distinct human AV sequences have been identified in human blood^[83]^. AV are found in many human tissues, but in longitudinal studies, nasal “colonization” seems to precede blood infection. AV shows a much higher genetic diversity than HIV, suggesting that AV is an “old” virus that invaded primates millions of years ago. So far, no disease could be associated with AV infections and some authors even consider AV to be a candidate for a beneficial viral infection that trains the immune system to deal non-aggressively with a viral infection in early development^[84]^. Some data from chronic viral infections seem to indicate that it is not primarily the cytopathology of the viral infection at the cellular level (which *Allelovirus* must induce during its replication cycle) which causes harm but an aggressive reaction of the immune system towards a “new, not yet adapted” virus that then leads to severe clinical symptoms^[85]^. 

### A respiratory virus with fecal excretion

At first glance, it might be surprising to find SARS-CoV-2 in the human gut. SARS-CoV-2 primarily replicates in the upper and lower respiratory tract. ACE2, the receptor for the virus on human cells, is not only widely expressed in the respiratory system but most prominently in gut epithelia. It should, therefore, not surprise us that up to 20% of Covid-19 patients also showed gastrointestinal symptoms, mostly in the form of abdominal pain, diarrhea and vomiting. Viral RNA was detected in feces, and its excretion frequently continued beyond the detection of SARS-CoV-2 RNA in respiratory secretions. A fecal-oral infection route was therefore initially considered, but epidemiological data failed to identify feces as a major source of infection. Evidence for intestinal replication of SARS-CoV-2 is equivocal^[86]^, and growth of infectious virus from feces in cell culture has failed, possibly because of the presence of an inhibitor in feces^[87]^. Currently, the fecal-oral infection route for SARS-CoV-2 is considered to be minor, if it exists, but that could not be anticipated since many animal coronaviruses, such as transmissible gastroenteritis virus, infect intestinal epithelia, causing nearly 100% mortality in piglets because it infects the stem cells in the crypts of the intestine. Another respiratory virus, avian influenza virus, also replicates in the gut, and large quantities of infectious viruses are excreted with avian droppings. The concern for fecal transmission of SARS-CoV-2 led to research into the presence of coronaviruses in sewage. Wastewater virology combined with PCR virus detection and later with metagenome sequencing opened a new field of public health activities^[88]^. The build-up of SARS-CoV-2 numbers could be detected in wastewater days before hospitals noted a rise in Covid-19 patients, providing early warning for public health authorities^[89,90]^. Wastewater virology is a diagnostic tool, not only for SARS-CoV-2. Recent observations of poliovirus increases in wastewater samples attracted attention to other enteric viruses that were from clinical data not considered to circulate at that scale in the human population of Western countries^[91]^. 

## CONCLUSION

At the tenth birthday of the HMP, I want to add some critical remarks to the gratulations against hasty conclusions that take the association of microbiome changes with disease as evidence for a causal link of the microbiome with human disease. To exclude microbiome changes as epiphenomena, i.e., consequences of pathological processes leading to disease without having itself a direct impact on the disease, there needs to be proof that intervention on the microbiome has an impact on disease expression. While such proof has been provided by FMT in *C. difficile* infections, the evidence is so far less clear with microbiome-targeting interventions in other gut conditions ranging from IBD to obesity and malnutrition. Adapted equivalents of Koch’s postulates are needed for human microbiome research to restrain unrealistically high expectations. 

As a second point, I want to draw attention to a third genome after “our second” bacterial genome, viruses. Calling this our third genome is probably as misplaced as referring to the bacteria as our second genome. In my opinion, it is questionable whether these microbes work to our benefit. Actually, evolution is driven by principles such as the desire of one bacterium to become two. Our body happens to represent a battle place in the ongoing fight of eukaryotes, prokaryotes, and viruses for a place in an ecosystem, be it the ocean, soil or our body. The use of the possessive pronoun “our” is misplaced here because it anticipates a selective choice of microbes from the environment for our benefit. Beneficial relationships between microbes and their hosts can develop, but many will remain neutral, some slightly negative, but not enough to justify investment into defense. Some microbes have very negative effects that can kill the host, as is vividly demonstrated when bacteria and viruses cause major epidemics. 

As the complex and scientifically impressive work on the link between the gut microbiome and obesity or malnutrition shows, the way towards a clinical application of microbiome interventions is a long and rocky one. When drawing attention to the human virome, I do not want to pretend that exploiting virome insights are low hanging fruit with respect to clinical application- the long and twisted story of phage therapy is a vivid illustration of difficulties in getting an attractive idea into clinical practice. My point is that phage research has demonstrated its scientific fertility several times in the past. At the 10^th^ birthday of the HMP, it is certainly time to extend the project to viruses. We need an analysis of the dynamic interaction of bacteria and viruses within the human host if we want to manipulate our microbiome to the benefit of human health. 

I hope that this perspective on various aspects of the human gut virome will motivate an increased focus on viruses in the future activities of the Human Microbiome Project. The author must confess that his view is influenced by the fact that he has worked as an industrial researcher for many years on various aspects of virology in the food industry. Human virome research might have many practical applications. In the end, I will only mention one approach for pandemic preparedness: Detecting and controlling emerging viral infections might possibly be easier at the level of virome screens in people living in close contact with wild animals in disturbed habitats or working at live animal markets rather than on wild animals as sentinels for zoonotic threats. Early warning and timely interventions might spare us from repeating the experience of the Covid-19 pandemic. 

## DECLARATIONS

### Copyright

© The Author(s) 2023.
